# Unmanned Aerial Survey of Elephants

**DOI:** 10.1371/journal.pone.0054700

**Published:** 2013-02-06

**Authors:** Cédric Vermeulen, Philippe Lejeune, Jonathan Lisein, Prosper Sawadogo, Philippe Bouché

**Affiliations:** 1 Unité de Gestion des Ressources Forestières et des Milieux Naturels, Université de Liège Gembloux Agro-Bio Tech, Gembloux, Belgium; 2 Office National des Aires Protégées, Ouagadougou, Burkina Faso; University of Illinois at Urbana-Champaign, United States of America

## Abstract

The use of a UAS (Unmanned Aircraft System) was tested to survey large mammals in the Nazinga Game Ranch in the south of Burkina Faso. The Gatewing ×100™ equipped with a Ricoh GR III camera was used to test animal reaction as the UAS passed, and visibility on the images. No reaction was recorded as the UAS passed at a height of 100 m. Observations, made on a set of more than 7000 images, revealed that only elephants (*Loxodonta africana*) were easily visible while medium and small sized mammals were not. The easy observation of elephants allows experts to enumerate them on images acquired at a height of 100 m. We, therefore, implemented an aerial strip sample count along transects used for the annual wildlife foot count. A total of 34 elephants were recorded on 4 transects, each overflown twice. The elephant density was estimated at 2.47 elephants/km^2^ with a coefficient of variation (CV%) of 36.10%. The main drawback of our UAS was its low autonomy (45 min). Increased endurance of small UAS is required to replace manned aircraft survey of large areas (about 1000 km of transect per day vs 40 km for our UAS). The monitoring strategy should be adapted according to the sampling plan. Also, the UAS is as expensive as a second-hand light aircraft. However the logistic and flight implementation are easier, the running costs are lower and its use is safer. Technological evolution will make civil UAS more efficient, allowing them to compete with light aircraft for aerial wildlife surveys.

## Introduction

Adaptive management and conservation of natural ecosystems require effective monitoring of biodiversity, including regular surveys of wildlife abundance [1]. In large African savannahs dominated by open vegetation and a flat landscape, aerial surveys with light aircraft remain the best alternative to count large mammals [2]. However, in some regions of Africa, such surveys are logistically difficult to implement due to the lack of appropriate aircraft and adequate fuel (aviation gasoline). Survey operations are also very expensive for most of the African states, which means that financial support from external donors is necessary to implement these operations [3]–[4]. The availability of external funds is often unpredictable, making long-term monitoring plans difficult.

As a consequence of these limitations, the time between successive surveys can often reach a decade and sometimes a quarter of a century in many protected areas [5]. During that time, some species could have disappeared [6]–[7] without any appropriate management action having been implemented.

The recent advent of UAS (Unmanned Aircraft Systems) in the scientific community raises the question of their possible use for future wildlife surveys [8]: can data from these pre-programmed flying machines soon replace the classic foot and aerial surveys of large mammalian fauna? The attempts to use this technology in the field of wildlife management have so far been limited to the occasional observation of animal species such as the bison (*Bison bison*) [9], the roe deer (*Cervus elaphus*) [10], the orangutan (*Pongo abelii*) [11], the alligator (*Alligator mississippiensis*) [12], marine mammals [13] or birds [14]. Could the elephant (*Loxodonta africana)* be added to this short list in a context similar to aerial sample surveys currently carried out by aircraft [15], [16], [17]? A first series of attempts has been made using a small UAS. The aim of this paper is to define the methodology to survey elephants with UAS and determine the flight parameters, as well as the animals’ reaction to the passage of the UAS.

## Materials and Methods

### Study Area

This study was implemented in the Nazinga Game Ranch (NGR) located in southern Burkina Faso along the international border with Ghana. It covers an area of about 940 km^2^. Its climate is essentially Sudanese and it lies in the southern Sudan savannah zone. Over the last decade, the mean annual temperature was 28°C and the mean annual rainfall ranged from 730 to 1,230 mm. The dry season begins in November and lasts until April or May. The wettest months are August and September, and generally very little or no rainfall is registered from December to March [18].

As part of the Sudanese regional center of endemism [19], the NGR is mainly covered with clear shrub and woody savannah (47.4%) characterized by *Vitellaria paradoxa, Terminalia spp.*, *Acacia dudgeoni*, *Gardenia erubescens*, *Pteleopsis suberosa*, in which the dominating perennial herbaceous species are *Andropogon spp.* and *Schizachyrium sanguineum* (Figure S1). The tree savannah, composed essentially of *Afzelia africana*, *Anogeissus leiocarpus* and *Lannea acida*, represents 25.4% of the total area [19].

### Material

**Figure 1:**
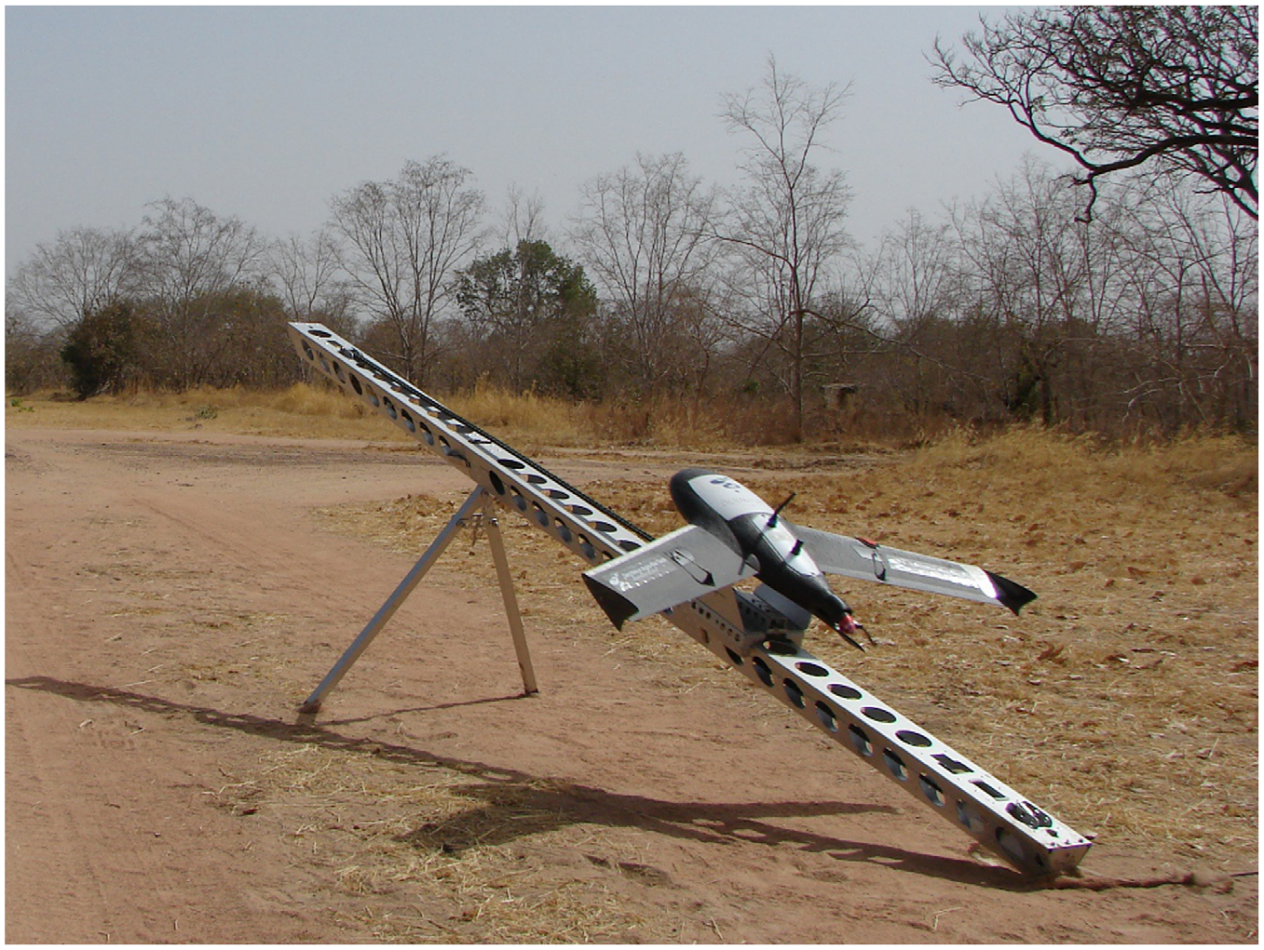
×100 on its launcher.

The Gatewing ×100 UAS (www.gatewing.com) (wingspan: 100 cm, weight: 2 kg, cruise speed: 80 km/h, flight height: 100 m –750 m, maximum flight duration: 40 minutes) was chosen for its silent electrical propulsion. It is equipped with a GPS, and an inertial measurement unit (IMU). These sensors determine the position as well as the altitude of the ×100 in flight. The GPS accuracy is a few meters, and the orientation angle (pitch, roll, twist) accuracy is 2 degrees (Klaas Pauli from Gatewing, personal communication). In order to prepare the flight plan using a specific software designed for the ×100 (QUICKFIELD™), flight characteristics (working area size and location, image overlap, height, take-off and landing points location, wind and landing directions) were recorded from a ground control station (GCS): a Yuma Trimble™ device. Then, another software (HORIZON™) was used to control the artificial altitude and heading reference system (AHRS) integrated in the electronic box (ebox) of the ×100. The UAS was catapulted with an elastic launcher system (Figure 1). The flight is fully automatic up to the landing and complete stop. In flight, the ×100 can keep contact with the GCS in a radius of about 5 km (flat land). After 15 minutes without contact with the GCS, the UAS moves automatically back towards its landing location. However, the user has the possibility to call back the UAS or interrupt the flight at any time. If the UAS crashes in a remote area, a VHF radio tracker with a range of 180 km inserted in the UAS can be used to locate and recover the UAS, thanks to a standard VHF radio-tracking antenna. The landing requires a flat strip 150 m long and 30 m wide clear of woods, termitary mounds or rocks. The operations after landing include the download of images from the SD card, flight data from the ebox, and GPS tracking.

Six 12 V nickel metal hybrid (Nimh) batteries delivered by the constructor were alternately used. Batteries were charged using a specific charger provided with the UAS. The charging time was about 1.5 to 2 hours according to the discharge level. The battery charger allows the use of both 240 V and 12 V (from car) power. If all batteries are charged, the time lapse between successive flights (from landing to next take off) was 25 to 30 minutes with 2 operators. This allowed 4 flights between 6 and 11 AM and 2 flights between 15 and 18 PM thus 6 flights per day in total.

The UAS was designed to fly up to Beaufort 6 wind speed (39–49 kph). The flight plan of the X100 was not affected by the wind or by the heat (up to 43°C in the shade). The UAS was operated at any time of the day even during the hottest hours. The risk of overheating is low due to batteries that can withstand heat while charging. The electric engine (250 Watts) is overpowered for the weight of the X100. The X100 proved its reliability in field conditions. The body part (fuselage+wings) is made of compact polystyrene and has to be replaced every 40 to 50 landings. Our body part was used a total of 35 times, in accordance with manufacturer specifications. In case of minor deterioration, the fuselage can easily be repaired with strong glue and a screwdriver. Small parts such as wingtips, elevon servos and rods, and pitot tubes can be changed if necessary. During this operation, a wingtips and a pitot tube were changed once. The camera suffered from an accumulation of rough landing and dust after its 45^th^ flight. We can conclude that the X100 was relatively reliable in rough terrain but the availability of a spare camera is recommended. A single trained person (training provided by Gatewing or its dealers) can operate the X100 but 2 trained operators are recommended to reduce the time for downloading, flight design and uploading operations.

An image overlap between 60 to 90% can be selected during the flight preparation. The UAS was equipped with a Ricoh GR3 still camera (10 megapixels, 28 mm Charged Coupled Device). Shutter speed (from 1/1600 to 1/2000) and camera sensor sensitivity ISO (from 100 to 400) were selected according to luminosity. Lenses were focused to infinity and focal was adjusted to 4.0. Images were taken automatically once the UAS reached its working area (transect or block). The UAS electronic box (ebox) is linked to the camera through a CB cable and sends a signal to start the continuous trigger of the camera. 8GB SD cards were used to record images. They allowed storing over 1100 10Mpixel shots. A 40-minute flight generates over 700 images (over 17.5 images per minute). The spatial resolution of the images obtained varied from 3 cm at a height of 100 m to 20 cm at a height of 600 m.

UAS flights over Nazinga were authorized by the “Agence Nationale de l’Aviation Civile” (ANAC) and the “Office National des Aires Protégées” (OFINAP) of Burkina Faso.

### Animal Reaction to the Passage of the UAS

Two tests of animal reaction to the passage of the UAS have been implemented. For each test, the UAS passed 10 times in the morning above the Akwazena pond at a height of 100 m along parallel lines 25 m apart from each other. A ground observer located close to the pond recorded all the animals seen as well as their reactions as the UAS passed.

### Animal Visibility

Five flights were conducted at flight heights of 100 and 300 m (Table 1) covering various habitats of the NGR (waterholes, woodland savannahs, forest galleries). A block of 1 km^2^ was covered by 10 to 12 parallel lines flown at a height of 100 m and strip-transect 10 km long, with a ground swath of 120 meters. After each flight, images were downloaded from the SD card of the camera onto a computer. Elephants were detected visually by displaying images on a laptop screen. The tests were also used to determine the visibility of different species. For one of the flights (13/02/2012), the presence and approximate location of animals were simultaneously recorded by ground observers and compared with the images taken from the UAS’s camera.

**Table 1:** Technical parameters and results of “animal visibility” flights.

Date	Location	Type of flight	Flight Duration (min)	Altitude (m)	Picture overlap (%)	Observed Elephants	Observed Buffon Kob	Observed Baboon
09/02/2012	Akwazena waterhole	Block count	17	100	70	3	No	No
13/02/2012	Akwazena waterhole	Block count	15	100	80	33 [33]	No [3]	No [17]
12/02/2012	Barka waterhole	Strip transect	22	100	60	7	No	No
12/02/2012	Barka waterhole	Block count	24	300	65	10	No	No
11/02/2012	Transect 22	Strip transect	33	100	60	28	–	–

Numbers in brackets correspond to animal simultaneously recorded by ground observers during the flight of 13/02/2012.

### Animal Count

Ten straight lines of 10 km were flown along the 4 transects used to carry out the annual foot count in NGR [18] between the 11^th^ and the 17^th^ of February 2012 at a height of 100 m. An overlap of 60% between images was selected. A total of 2732 images were recorded during these flights. Four independent operators counted animals from images displayed on the same laptop screen. For each group observed on images, animals were discriminated according to 2 classes: (i) adult, (ii) sub-adult and calf. The reference of the picture on which the animals were observed was also noted in order to cross-check the different counts.

### Data Analysis

Counting data were analyzed using the Jolly method 2 for unequal sampling count.

This method is commonly used to analyze strip sample aerial counts performed with light aircraft [17]. The density estimation (eq 1) corresponds to the ratio between the number of encountered animals to the total sampling strips area (width × length).
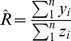
(1)with 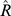 is the ratio of animals counted to sampled area (Density) [animals.km^−2^],


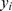
 is the number of animals counted in the sample unit,


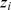
 is the area of the strip [km^2^]


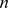
 is the number of sample unit (strip) in the sample.

The sampling error estimation is given by equation 2.
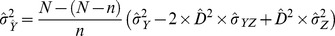
(2)with/is the variance of the estimated population,


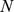
 the number of sample unit in the population,


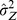
 is the estimated variance of Z,


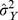
 is the estimated variance of Y,


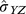
is the estimated covariance.

## Results

### Animal Reaction to the Passage of the UAS

Table 1 shows the total number of animals (3 mammal species) present along or in the pond during the 2 tests. No flight or warning behavior was recorded for any of the species.

### Animal Visibility

**Figure 2:**
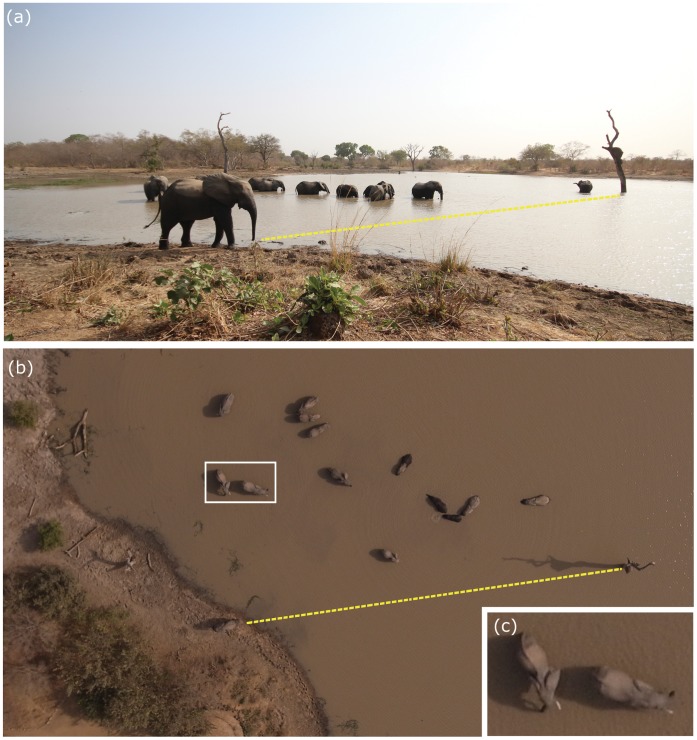
Photo of elephants bathing in the Akwazena pond. (a) Ground image and (b) Aerial image of an elephant group bathing in the Akwazena pond. The dotted yellow line on both images links to two referenced features (an elephant and a tree). Picture (c) is an enlargement of part of the aerial picture.

**Figure 3:**
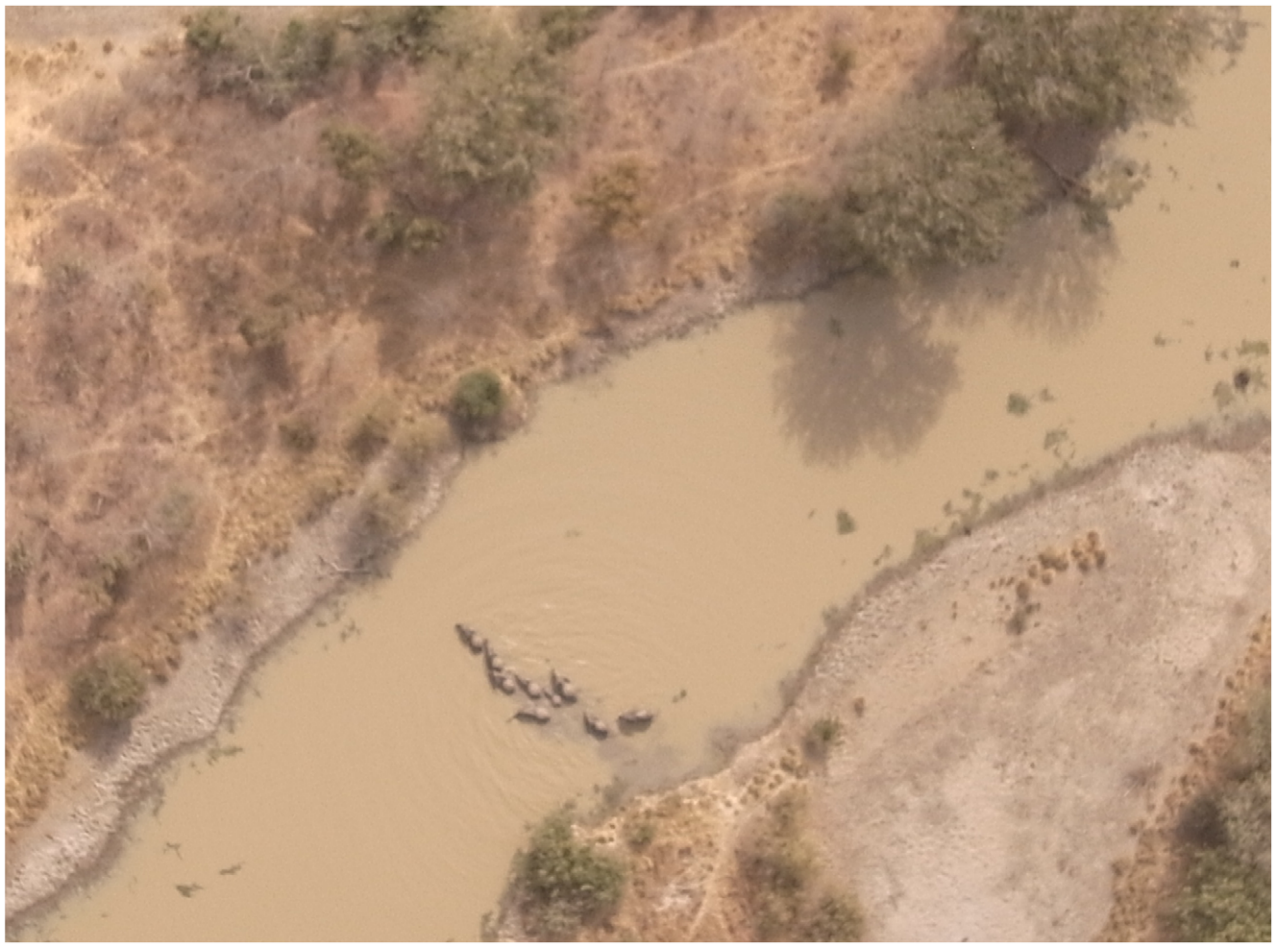
Aerial photo of elephants taken at a height of 300 m.

The first significant result of this study regarded animal visibility. The 5 flights (Table 1) demonstrated that the elephant is easily visible at an altitude of 100 m. For example, a group of 13 individuals bathing and 1 on the bank of the water body were photographed simultaneously from the ground and from the UAS at an altitude of 100 m (Figures 2A and 2B). Each individual is clearly identifiable on the aerial image. In addition, animal enumeration was easier from the aerial image. Elephants remained discernible up to an altitude of 300 m in his natural habitat (Figure 3).

However, the Buffon kob (*Kobus kob kob*) was difficult to observe on the images. The baboon (*Papio anubis*) could not be formally detected.

The elephants were then searched for on every available image set. Elephants were easily discernible at a height of 100 m but not at a height of 300 m. From images, it was possible to discriminate the elephant group’s composition: adults, sub-adults and calves.

The analysis of these images enabled us to conclude that the observation and thus the count of elephants in the Sudano-Sahelian savannah ecosystem (Figure 2A, 2B and 2C) is possible on such images.

### Animal Count

The succession of images acquired along the flight lines recreated classic strip-transect aerial count conditions. Image count was carried out at a mean rate of 27.81 images per minute. Finally, cross-checked counts revealed that 34 elephants were present inside the sampling strips. Independent counts showed that, on average, 14.7% elephant were missed (Table 2). Combining individual counts with cross-checked counts into duos of independent observers lead to an average missing rate of 7.8%. The estimated density was 2.47 elephants km^−2^ with a coefficient of variation (CV%) of 36.10% (Table 3). We assume that this dataset contains very few observation, and thus, do not fully comply with the assumptions of normality of the parent population. Those results have, therefore, only a demonstration value.

**Table 2:** Operator effect on elephant counts from images taken from the UAS.

Operator	Time of analysis	Nb of elephants	
	minutes	counted	missed
1	108	33	1
2	91	29	5
3	76	23	11
4	118	31	3

**Table 3:** Elephants seen along transects.

Flight date	Transect nb	Nb of images	Number of elephants	Transect length (km)	Sample area (km^2^)
10/02/2012	L23	297	0	11.98	1.474
10/02/2012	L24	271	0	10.89	1.340
11/02/2012	L22	309	34	12.49	1.536
11/02/2012	L25	229	0	9.21	1.132
11/02/2012	L26	188	0	7.67	0.944
13/02/2012	L21	321	0	12.94	1.591
17/02/2012	L22	319	0	13.18	1.621
17/02/2012	L23	299	0	12.61	1.552
17/02/2012	L24	272	0	11.65	1.433
17/02/2012	L25	227	0	9.47	1.165
		2732	34	112.09	13.788

## Discussion

### Animal Reaction as the UAS Passed

The absence of animal reaction as the UAS passed is remarkable and indicates an absence of animal disturbance. But this absence of reaction could appear as a potential drawback in the future because animals are more easily visible when they are moving than when they are standing. If we are able to mount a video camera on an UAS, the recording of animal movements will improve their detectability.

### Animal Visibility

Adult elephants’ visibility is excellent at a height of 100 m and possibly at 300 m. The detection of elephant calves was facilitated by their close proximity to the adults. We cannot avoid a certain amount of uncertainty in the count of calves. The use of computer recognition algorithms is worth investigating for the detection of adult elephants.

This information is important because it has an impact on the size of the sampling area. At a height of 100 m, the width of the sample strip is 120 m according to the camera used. These images can even be used to determine the age of the elephants by measuring back lengths, considering the pixel size (from 3 to 10 cm) [20], [21]. The detectability of other species was disappointing. None of them were detectable by a rapid naked-eye image analysis on a laptop screen.

### Animal Count

The results show that an aerial sampling count is possible with a small UAS. According to the survey protocol (one pass strip-transect, height of 100 m, ground swath of 120 m) double counting along the same strip transect is impossible due to UAS speed (80 km/h). In contrast, the mean time between 2 successive flights (45 minutes) leaves the possibility for an elephant to shift between 2 transects separated by 1.5 km. The group composition is used to discriminate herds. In this case, all elephants were observed along the same transect and there is no possibility of double count. Experienced observers are required to analyze images. Missing a group has important consequences on the final estimate especially when elephants are few in number. Counts made by a pair of independent observers are therefore recommended and the results should be cross-checked. In addition, use of two observers can be used to estimate detection probabilities [22].

### Recommended Survey Protocol

Based on the first tests, we recommend the implementation of elephant surveys at a flight height of 100 m (ground swath: 120 m), transects of maximum 10 km spaced every 1.5 km (sampling effort of 9.6%). A height of 100 m is a good balance between the detection (2560 pixels/elephant at a height of 100 m) and the swath width. A maximum of 6 transects of 1.2 km^2^ can be implemented every day (4 in the morning and 2 in the evening). The transect length of 10 km is a result of software constraints. The software was originally designed to cover blocks rather than transects (the operator cannot design the interval width between successive lines forcing to flights to cover one transect at a time). This constraint will be minimized once the software is redesigned to fly 2 successive 15 km transects.

Beyond 5 km, the Gatewing ×100 loses contact with the control station but comes back automatically after 15 minutes of flight without contact with the ground control station. It is thus recommended that there be enough airstrips to cover an area of a multiple of 75 km^2^ (6 transects, thus 5 intervals of 1.5 km × 10 km).

### Perspectives

The use of UAS such as the ×100 opens interesting possibilities for counting elephants. The technology is sufficient to count African elephants in savannahs: flight implementation is easier (very short airfield), safer (no operators on board) and the UAS is reliable in very rough conditions. The UAS flights require civil aviation authorization. However, the main drawback of the Gatewing ×100 is its low autonomy. Unlike a light aircraft, this small UAS cannot cover large areas in a minimum of time (4 to 6 hours per flight). If some UAS cost as much as an aircraft, the logistic (only one 4×4 car) and the running costs of the UAS are lower (Table 4). However, the cost per area covered (km^−2^) is almost 10 times higher than that of an aircraft. Also, the characteristic shape and biometry of elephants on the nadir images allow us to consider use of computer recognition algorithms.

**Table 4:** Running cost of UAS vs aircraft (in Euro). Human resource cost is not included.

	Flight hour/day	Flight cost/hour	Cost/day	Area (km^2^)/day	Cost/km^2^
UAS	6	71	426	7.2	59.17
Aircraft	6.5	400	2600	384	6.77

×100 UAS running costs have been calculated as follows: It was assumed that the body of the UAS must be replaced every 40 flights. Each flight duration was estimated in mean at 0,6 hours, totaling 24 hours flight for a body of 1500 € thus 62.5 € per flight hour. Camera repair cost was estimated at 100 € per body life (24 hour flight) thus 4.17 Euro per hour. Battery recharging was assuming free. Antennas and servo rods replacement have been estimated each at 2 € per hour.

Other UASs than the Gatewing ×100, whether electrically or liquid fuel powered should be considered in order to improve the autonomy and the payload. A larger autonomy will ensure a control range of a few hundred km where as more important payload will allow the use of a camera with a higher resolution and thermic cameras. Such UASs can truly become an alternative to the use of light aircraft in African wildlife surveys.

## Supporting Information

Figure S1 **Clear shrub and woody savannah of Nazinga Game Ranch.** Aircraft costs include the aircraft rental (250 Euro/hour) and the aircraft fuel at 3 Euro per liter (in West Africa). A suitable aircraft consumes about 50 l per hour.
(TIF)
