# Supplementary figures and images for: Unmanned Aerial Survey of Elephants
Source: PLoS One. 2013 Feb 6;8(2):e54700. doi: 10.1371/journal.pone.0054700 (PMC3566131; doi:10.1371/journal.pone.0054700)

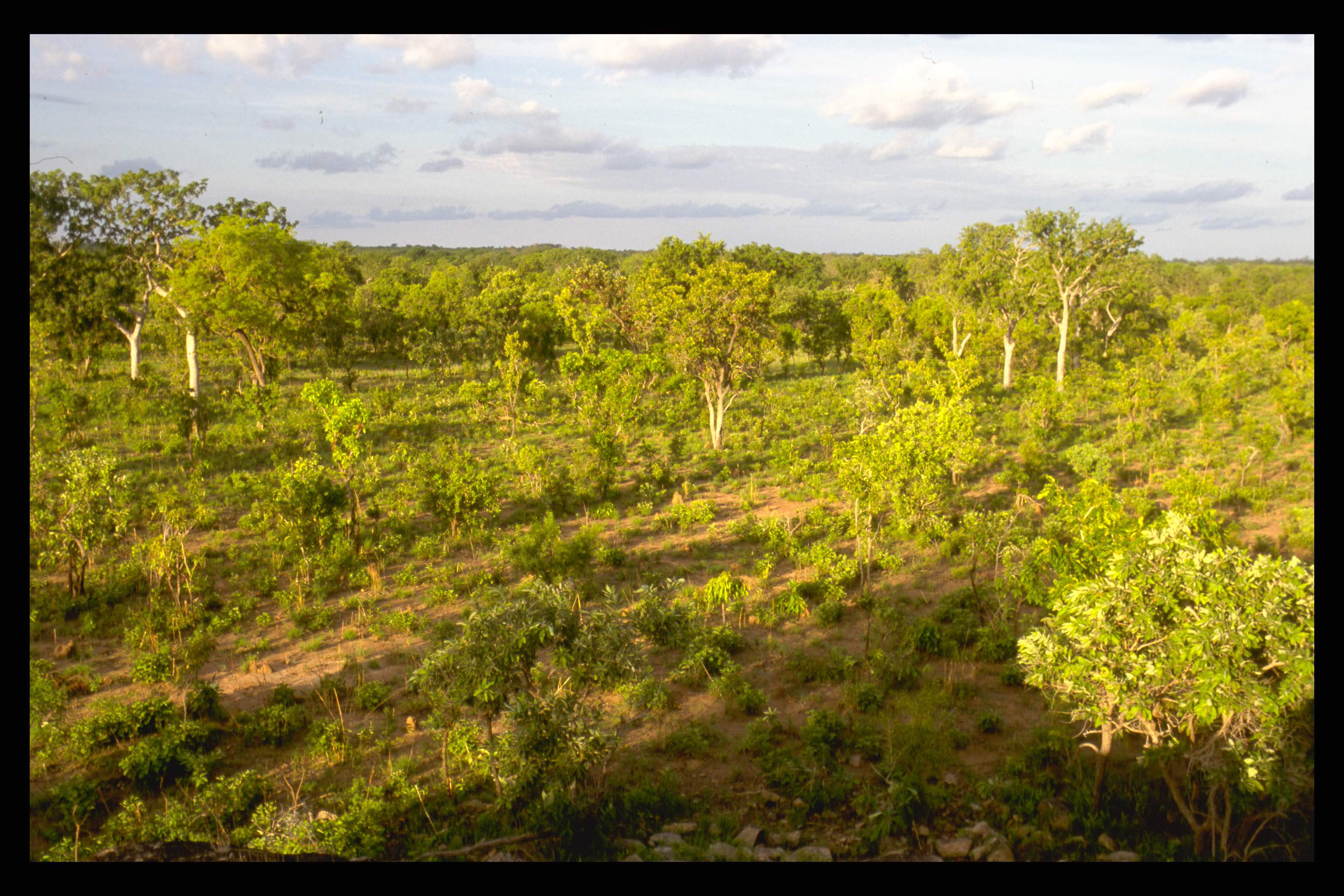

Supplement: Figure S1 — Clear shrub and woody savannah of Nazinga Game Ranch. Aircraft costs include the aircraft rental (250 Euro/hour) and the aircraft fuel at 3 Euro per liter (in West Africa). A suitable aircraft consumes about 50 l per hour. (TIF) [file pone.0054700.s001.tif]
